# Psychic suffering and depression in black children and adolescents: systematic review and meta-analysis

**DOI:** 10.1590/1414-431X2020e10380

**Published:** 2021-07-16

**Authors:** J.M.M. Landim, M.L. Rolim, D.M. Christofolini

**Affiliations:** 1 Centro Universitário FMABC, Santo André, SP, Brasil; 2 Universidade Federal do Cariri, Juazeiro do Norte, CE, Brasil

**Keywords:** Depression, Children, Adolescents, Black person

## Abstract

Depression is a common disorder in the population, but some people are more vulnerable to this condition. Groups at higher risk of developing psychic suffering include black children and adolescents living in vulnerable socioeconomic conditions. This study aimed to analyze race and life conditions as determinants of depression in children and adolescents. This was a systematic review with meta-analysis. The study sources were MEDLINE Ovid, Web of Science, Latin American and Caribbean Health Science Information database, Science Citation Index-Expanded, PubMed, EMBASE, and Scopus. The following keywords were used: Child, Adolescent, Stress, Psychological, Depression, and African Continental Ancestry Group, using the logical operators AND and OR. The general criteria were observational studies published in the last 20 years. Language was not restricted to avoid possible bias in the selection of articles. Studies with a high risk of bias were excluded. General analysis was conducted with RStudio 3.0 software using odds ratio analysis with a 95% confidence interval and 0.05 significance level. We firstly found 654 studies, of which 18 met the criteria and were included in this review. Race and life conditions were determinants of depression in children and adolescents, with a negative impact for the black population.

## Introduction

Depression is a psychological condition affecting around 6% of the world's population. Factors like socioeconomic variables, health, and race may contribute to a higher level of predisposition to the disease (1). In children and adolescents, these predictors may be even stronger and have significant impact on mental health, especially in black individuals (2).

Low educational and income levels and use of drugs are some of the points that should be considered. When these factors are associated with race-based prejudice, the association with mental dysfunctions, including depression, is stronger (3). These variables must be analyzed so that intervention strategies can be determined.

Factors that have longitudinal prejudice have interpersonal and transactional sides. Therefore, not only personal but also external factors may influence the process of mood disorders (4). The presence of other related diseases, such as infections, has a cause-effect relationship with psychic suffering (5).

Based on these effects, we need to take into consideration several conditions and narratives associated with children, adolescents, and their families that, in general, have a higher risk of developing mental diseases like depression. Thus, analysis of these factors is fundamental to determine strategies to be adopted in this group.

Hence, are race and socioeconomic conditions significant determinants of depression in children and adolescents?

The relationship between race and depression is well-known, but a meta-analysis of published results is lacking. In addition, the inclusion of possible associated factors in the analysis allowed us to verify whether there is a significant impact for children and adolescents that could generate life-long changes. Thus, this global analysis, involving samples from different groups, has a gap that needs to be filled.

This study aimed to analyze whether race and life conditions are determinants of depression in children and adolescents.

## Material and Methods

A systematic review with meta-analysis was carried out according to the recommendations of the PRISMA protocol (identification number 259534) (6).

Qualitative and quantitative epidemiological studies were included to allow the calculation of the effects of each analyzed variable/group. The general inclusion criteria were observational studies published in the last 20 years. Language of publication was not restricted to avoid possible bias in the selection of studies. Studies with a high risk of bias were excluded.

We included studies with black children and adolescents from Portuguese-speaking countries for an analysis of the potential risk factors and determinants of depression. We assessed variables that could be considered sources of influence according to the PVO acronym (Population, Variables, and Outcomes), as follows: P: Portuguese-speaking black children and adolescents; V: socioeconomic and skin color vulnerability; O: depression.

We removed studies that discussed these aspects in a sporadic way so that they did not influence the results. For effect determination, the primary outcomes were depression and general psychic suffering and the secondary outcome was other mental disorders. The search was performed in MEDLINE Ovid, Web of Science, Latin American and Caribbean Health Science Information, Science Citation Index-Expanded PubMed, EMBASE, and Scopus electronic databases.

The search strategy used in all databases was based on previous searches and included the words: (Child OR Adolescent) AND (Stress, Psychological OR Depression) AND (“African Continental Ancestry Group”).

Two reviewers and authors worked independently to extract predetermined information. In the event of a discrepancy, a third reviewer was consulted for data determination and inclusion or removal of the article. The search was conducted in the first semester of 2020 (March 25 to June 26). Restricted articles were obtained through contact with the researcher, and other studies were used to complement the discussion and general idea of the systematic review. A final search in related sources, such as preprints or partial data, was carried out to determine the final sample of studies.

The information collected from each study was: year of publication, country of origin, authors, method, number of subjects, inclusion and exclusion criteria, screening forms, and baseline characteristics of subjects.

For the analysis of outcomes, we collected the number of subjects and mean and standard deviation of descriptive variables. Two authors worked independently in the process of searching and selecting studies using the Rayyan software (https://www.rayyan.ai/). In case of disagreement between the researchers, a third reviewer was consulted to determine whether or not to include the study.

Analysis of the effects was done according to dichotomous or continuous data, based on the characteristics of the selected studies and using a 95% confidence interval (CI). Whenever possible, we searched for absent data and the multiple imputation method was used for missing cases.

Heterogeneity refers to the extent to which studies in a review differ from each other. We chose the most homogeneous studies for a better determination of effects. Studies with I^2^ above 50% were considered heterogeneous. The analysis was done with RStudio 3.0 software (https://cran.r-project.org/bin/windows/base/) using the meta package and odds ratio analysis with a 95%CI and 0.05 significance level. Summary estimates were calculated for each study such as sample percentages, means, standard deviations, and measures of association when available.

## Results

**Figure 1:**
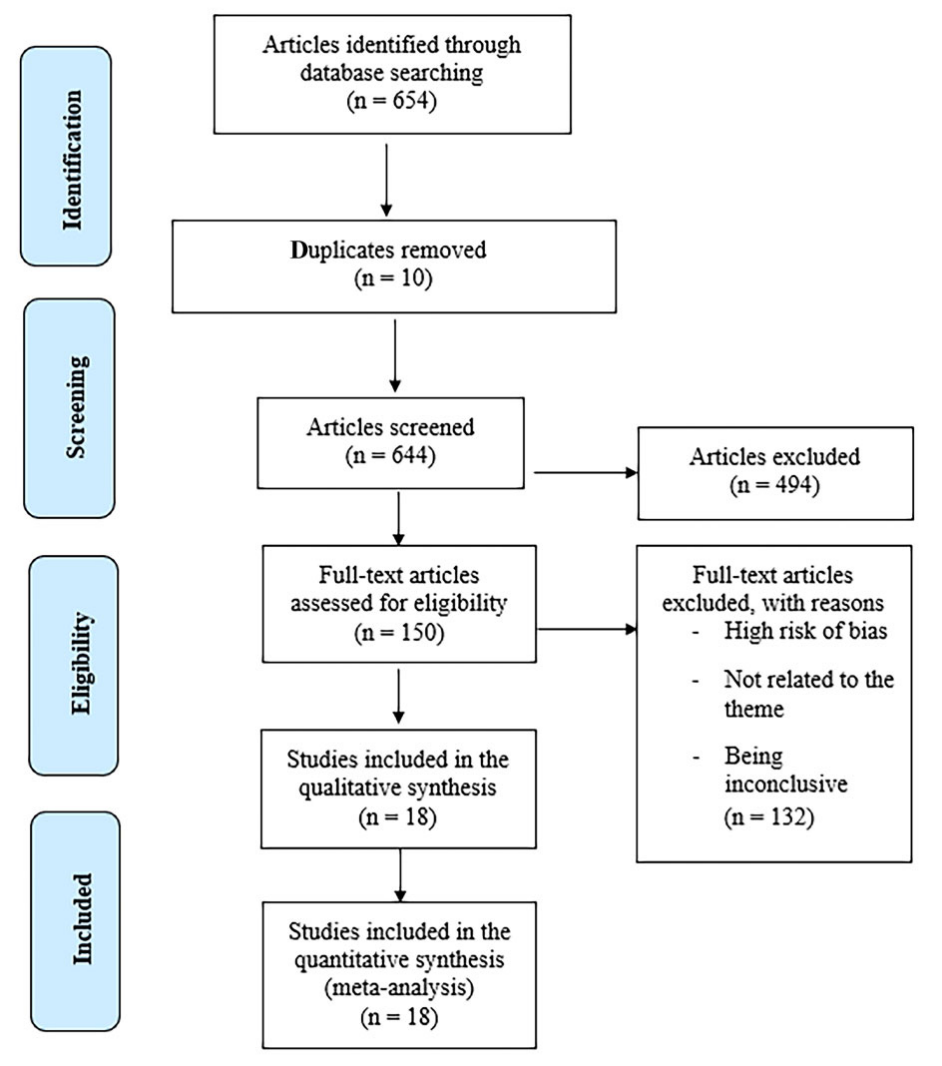
PRISMA flow diagram of study selection.

We firstly found 654 studies, of which 18 met the criteria and were included in the review. The flowchart in Figure 1 summarizes the study selection. With the use of the Rayyan tool, 132 studies were excluded, as they were not within the scope of our study.

Table 1 shows the main characteristics of the studies, including authors, objective, method, and main outcomes. Most of the selected studies were observational and the outcomes point to some of the main risk factors that may be related to mental changes in the population studied.

**Table 1:** Synthesis of the selected studies.

Author (reference)	Objective	Method	Main outcomes
Rotheram-Borus et al. (1)	To analyze depression through the presence of comorbidities	Cohort	Alcohol abuse, income, and diseases like HIV influence depression development
Mellick et al. (2)	To analyze the invariance of classifications of depression symptoms in African American, Hispanic/Latin, and Caucasian adolescents that are psychiatric patients	Cross-sectional	Ethnicity is a risk factor for mental diseases
Hazzard et al. (3)	To examine the association of mental disorders in adolescents with differences of skin color/race	Longitudinal	The early identification of these cognitions and early intervention may help reduce the risk of depression in the young adult age.
Davis et al. (4)	To examine the paths between victimization, depression, academic performance, and problematic consumption in adolescents	Cross-sectional	Results are discussed regarding the prevention interventions for problematic consumption, as well as screening for adolescent depression
Earnshaw et al. (5)	To analyze the problems related to stigma, depression, and use of substances among young people	Cross-sectional	The internalized stigma is associated with a higher risk of depression, and the associative stigma is associated with a higher risk of depression and problems related to substance use
Weller et al. (7)	To estimate the national prevalence of comorbidities for black, Hispanic, and white adolescents separately and compare the prevalence of comorbidities among adolescents with and without depression	Cross-sectional	This study found important differences in the prevalence of comorbid psychiatric conditions by race/ethnicity. Results highlight the need of interventions directed to black and Hispanic adolescents with depression that are simultaneously treating anxiety and behavioral problems.
Hussen et al. (8)	To analyze social capital, depressive symptoms, and viral suppression of HIV among black, gay, bisexual, and other kinds of adolescents that have sexual intercourse with men living with HIV	Cross-sectional	Socioeconomic factors are a risk for the development of mental comorbidities
Kilpatrick and Taylor (9)	To assess the importance of perceived prejudice in a multiethnic sample of study participants with and without physical disability	Cross-sectional	Results show race as a source of prejudice and health risk
Mowbray et al. (10)	To analyze race as a depression determinant	Cross-sectional	Race is a risk factor for depression
Assari et al. (11)	To investigate the association between family income and risk of major depressive disorder (MDD) in black young individuals, based on ethnicity, gender	Cross-sectional	Results suggest that ethnicity and gender influence how socioeconomic resources, like income, are associated with risk of MDD
Bromberger et al. (12)	To analyze childhood socioeconomic circumstances and depressive load of symptoms throughout 15 years of follow-up in midlife	Cohort	Socioeconomic factors can be mental health determiners
Youssef et al. (13)	To examine: 1) if there is a dose-response relation between trauma and depressive symptoms; 2) if the initial trauma affected European Americans (AE) and African Americans (AA) in a similar way; and 3) if resilience reduces the trauma effect	Cross-sectional	Even though the adverse experiences have been significantly associated with the depression severity of the dose-response form, a higher resilience mitigated the impact of childhood adversities in depressive symptoms in young adults
Jesse et al. (14)	To analyze the role of routine events in depression	Cross-sectional	Impact on mental health of being young and black is significant
Montalvo-Liendo et al. (15)	To analyze the ethnical-race variation for depression	Cross-sectional	Even though it is important to assess the mental health needs of all women who are victims of abuse, these results show an additional need of Latin women, those with low income and high exposure to violence.
Mereish et al. (16)	To examine ethnicity as the moderator of the mediator effects of self-esteem about the relationship between prejudice and depressive symptoms	Cross-sectional	Important ethnic differences are highlighted among black men
Duffy et al. (17)	Describe the development, feasibility, and acceptability of a novel preventive intervention for depression in African American girls living in urban poverty	Clinical trial	Results indicate that the viability of a novel preventive intervention for depression in African Americans was weak, considering that satisfaction and usability were high. Future directions to test the efficacy are discussed
Mrug et al. (18)	To explain the differences in depressive symptoms among African American and European adolescents	Cross-sectional	The family's socioeconomic factors reduced this difference in 29%; all the risk factors were reduced in 88%. Exposure of adolescents to violence, antisocial behavior, and low connection to school, as well as lower education of parents and parenthood quality, appeared as significant mediators of the differences between groups in depressive symptoms
Smith et al. (19)	To examine to what extent the positive mental well-being of adolescents and depressive symptoms vary between the ethnic groups and to examine prospectively if social support is a protection against low/poor well-being and depression.	Longitudinal	Bangladeshi and Black African adolescents in East London may have a positive advantage in mental health compared to their white counterparts from the United Kingdom, even though social support does not completely explain the results.

The GRADE system indicated that there was a high certainty of evidence in the studies due to the small bias level. Table 2 provides the joined bias risk according to GRADE 6 (http://www.jclinepi.com/content/jce-GRADE-Series) assessment tool.

**Table 2:** Bias between studies according to the GRADE system.

**Certainty assessment**	**Depression**
Number of subjects (studies) Follow-up	18 observational studies
Bias risk	Not severe
Inconsistency	Not severe
Indirect evidence	Not severe
Inaccuracy	Not severe
Publication bias	Very strong association
Overall certainty of evidence	High
**Summary of Results**	
With black adolescents and children	Present feature/absent feature
Relative effect (95%CI)	Non-calculable
Potential absolute effects	
Risk with black adolescents and children	Low 60/1000
Risk difference with risk factors	Not related

CI: confidence interval.

**Figure 2:**
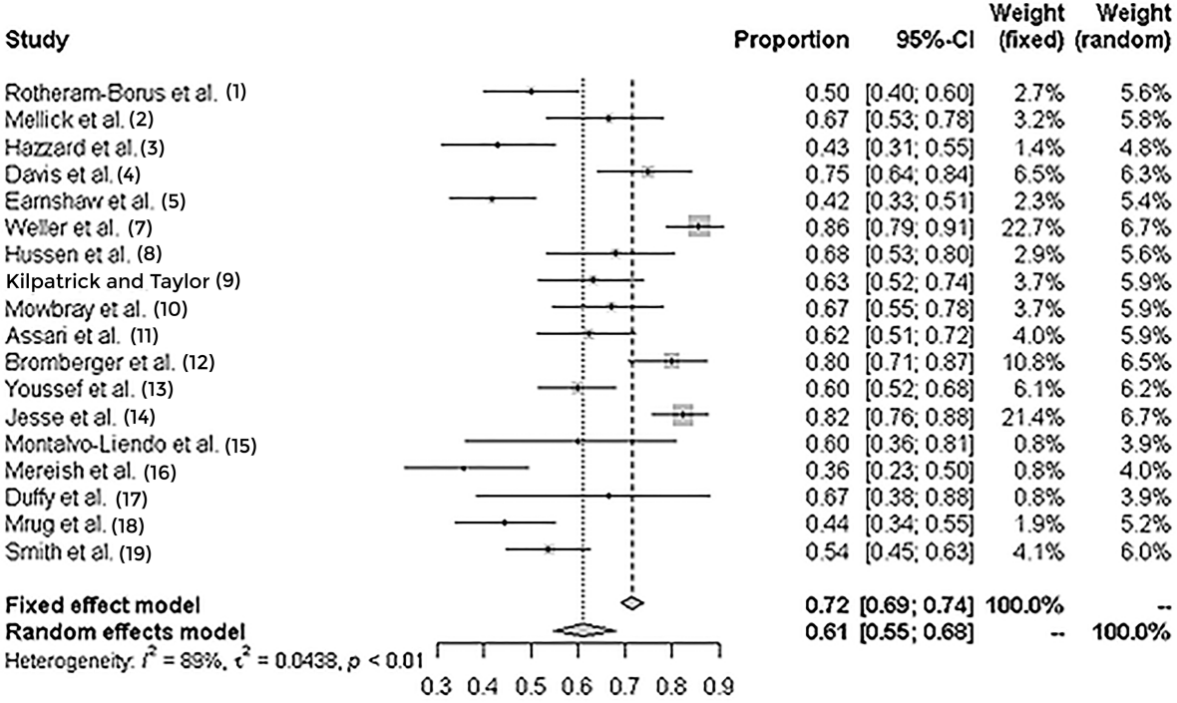
Forest plot of the meta-analysis of ethnicity as a determinant of depression in black children and adolescents.

Figure 2 shows the calculation of the meta-analysis, a combination of studies on the theme, in which it was possible to determine an effect size of 0.61 with 95%CI (0.55-0.68), which attests that even considering different contexts, the rate of depression in black populations was quite prevalent.

**Figure 3:**
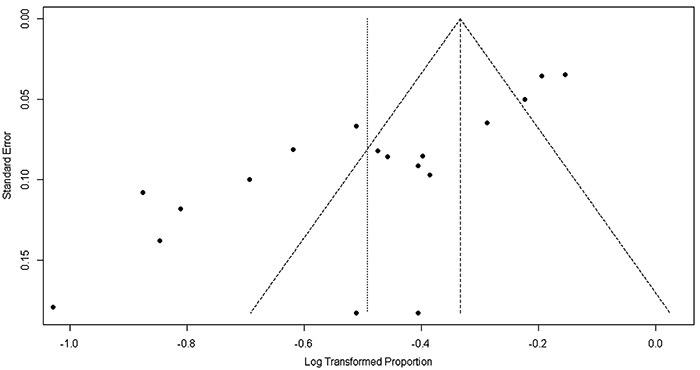
Funnel plot (publication bias).

Figure 2 presents the results of the meta-analysis on ethnicity and Figure 3, the publication bias analysis of the studies. Both analyses showed that black children and adolescents in vulnerable social conditions tend to present a strong association (0.73; P<0.001) with depression and mental suffering. Table 2 also presents a summary of the outcomes of the analyzed studies.

## Discussion

The results showed a significant association between ethnicity and depression with a P-value <0.001. A study carried out by Weller et al. 7 reported that depression and anxiety were present in 47% of the individuals. This fact influenced the behavior adopted regarding health, such as lower use of disease preventive methods, and resulted in a larger number of potential diseases.

For Hussen et al. 8, social capital and social relationships determine the vulnerability to certain conditions, like low acquisition of health inputs, which leads to a higher prevalence of some diseases in the black population, such as the human immunodeficiency virus (HIV) infection. This would be an important predictive factor for the ‘depression’ outcome especially regarding adolescents.

Prejudice issues, which are very common in this context, are also associated with health status. Racism was reported by Kilpatrick and Taylor 9 as an important risk factor for poor health. However, for Mowbray et al. 10, race is independently associated with other sociodemographic variables, because black people with a stable economic condition tend to have an increased risk of developing depression.

In addition, Assari et al. 11 reported that family plays an important role, especially for children and adolescents. Family support in the situations experienced during this period is essential to determine the risk of mental diseases like anxiety and depression. The average age of 15 years has been more associated with a group of economic stressors and limited social resources that result in consequences, including mental alterations 12.

Ethnic and racial adversity in childhood, including abuse and varied traumas, increase the possibility of depressive symptoms. These events generate resilience levels that affect mental health, especially in the black population 13,14. Gender issues should also be assessed as risk factors for psychic suffering: women are more prone to the development of depression that is also associated with low income and exposure to violence 15–17.

Exposure of adolescents to violence, antisocial behavior, low school attendance, low educational level of parents, and quality of parenthood appeared as significant mediators of differences in depressive symptoms 18. Lower levels of social support are associated with decreased well-being and higher rates of depression in children and adolescents 19.

Based on the discussion and results, we found that race and associated life factors influenced the development of depression in children and adolescents. Some populations, such as individuals from low-income countries, would be more prone to mental health disorders, not only because of prejudice, but also because of poor socioeconomic factors that contributed to the process.

The main study limitation was the low number of publications regarding mental health in black individuals, especially children and adolescents.

### Conclusions

Race and life conditions are determinants of depression in children and adolescents, with a negative impact in the black population. Social narratives could be investigated for a qualitative determination of feelings and revelations regarding the findings - this study found a statistical association - and how these people feel regarding life aspects should also be taken into consideration.

Black individuals from Portuguese-speaking countries with low socioeconomic levels live in a vulnerable environment that contributes to psychic suffering, and this should be studied in more detail to formulate strategies to reduce the impact on these people.
